# Clear Cell Cancer of the Uterine Corpus: The Association of Clinicopathologic Parameters and Treatment on Disease Progression

**DOI:** 10.1155/2011/628084

**Published:** 2011-11-30

**Authors:** Joyce Varughese, Pei Hui, Lingeng Lu, Herbert Yu, Peter E. Schwartz

**Affiliations:** ^1^Division of Gynecologic Oncology, Department of Obstetrics, Gynecology, and Reproductive Sciences, Yale University School of Medicine, 333 Cedar Street, P.O. Box 208063, New Haven, CT 06520, USA; ^2^Department of Pathology, Yale University School of Medicine, 310 Cedar Street, New Haven, CT 06520, USA; ^3^Yale School of Public Health, Yale University, 60 College Street, New Haven, CT 06520, USA

## Abstract

This paper presents a single-institution experience regarding the clinicopathologic features and treatment strategies used in uterine clear cell cancer (UCC), a rare, aggressive histologic subtype of uterine cancer with poor prognosis and discusses parameters associated with progression-free survival (PFS) and overall survival (OS). A retrospective chart review was performed on all patients (*n* = 80) diagnosed with UCC and treated between 1994 and 2009 at a single academic institution. Data on demographics, FIGO stage, treatment regimens, and recurrences were collected. Patients with early-stage UCC had an excellent survival regardless of adjuvant therapy. Advanced-stage patients had a worse survival. Vaginal apex brachytherapy was associated with an increased OS (*P* = 0.02) but not PFS (*P* = 0.10). The use of platinum-based chemotherapy in combination with vaginal apex brachytherapy did not significantly improve survival. Innovative therapies still need to be identified for this uncommon uterine cancer.

## 1. Introduction

Endometrial cancer is the most common gynecologic cancer in the United States, with 43,470 new cases and 7,950 deaths estimated in 2010 [1]. The incidence of endometrial cancer is greater than that of all other female genital tract malignancies combined [1]. Clear cell carcinoma of the uterine corpus (UCC), a rare subtype accounting for 1–6% of uterine cancers, is characterized histologically by the clearing of tumor cell cytoplasm [2–5]. Patients with UCC are more likely to present with higher-stage disease than those with endometrioid histology, and clear cell histology has been considered a poor prognostic factor [2, 6]. Comprehensive surgical staging is recommended in women with UCC, given the high error rate with clinical staging [7]. Aggressive, multimodal treatment (including surgery, chemotherapy, and/or radiation therapy) is usually recommended as compared to type I endometrial cancers. Due to the rarity of UCC, there are no prospective studies evaluating these treatments solely in women with UCC [4].

Compared with endometrioid and serous carcinomas, little is known about the molecular pathways and immunophenotypic profile involved in UCC. Studies have confirmed that UCC is genetically distinct from endometrioid cancer [8]. Clear cell tumors show similar gene expression profiles regardless of organ of origin [8, 9]. Type II endometrial tumors tend to display p53 mutations, compared to Type I cancers [2].

The goal of this paper was to present the clinicopathologic features and treatment strategies used in UCC and determine what parameters are associated with progression-free survival (PFS) and overall survival (OS).

## 2. Materials and Methods

A retrospective chart review was performed on all patients with the diagnosis of UCC from 1994–2009 who were treated at the Yale-New Haven Hospital (YNHH). A YNHH Tumor Registry search under the search terms “endometrial cancer” and “clear cell” identified 80 patients with UCC.

Medical charts, including admission and discharge notes, as well as surgical pathology reports and treatment records (chemotherapy and radiation) were reviewed, and epidemiological data (age at diagnosis, ethnicity, gravity, and parity), clinical data (past medical history, menstrual history, smoking history, hormone or tamoxifen use, personal or family history of other malignancies, body mass index (BMI)), and pathologic and histological data (stage, lymphovascular space involvement, positive pelvic washings, involvement of polyps, lymph node metastases, depth of myometrial invasion) as well as survival data (disease-free and overall survival) were extracted. All pathology and cytology specimens had been previously reviewed by gynecologic pathologists at YNHH. Seventy-six patients were staged using the International Federation of Gynecology and Obstetrics (FIGO) 1988 operative staging system for endometrial cancer. The remaining 4 patients had palliative treatment only.

Long-term follow-up data were censored at date of last followup. PFS was calculated from date of diagnosis until date of recurrence, death, or last followup. OS was calculated from date of diagnosis until death or date of last followup. Cox proportional hazards multivariable models and Kaplan-Meier test were used for survival analysis. A *P* < 0.05 was considered statistically significant. Statistical analyses were performed using SAS 9.2 (SAS Institute, Cary, NC). This research was approved by the Human Investigation Committee at Yale University School of Medicine (HIC#0804003674).

## 3. Results

A total of 80 patients with UCC were identified. Their mean age at diagnosis was 67 years (range 43 to 91 years) (Table 1). Most patients were Caucasian (86.3%, *n* = 69). Medical comorbidities at the time of diagnosis included hypertension (57.5%), diabetes (27.5%), and coronary artery disease (17.5%). Parity was known for 67 patients (83.8%), and of these patients, 95.5% were parous. Eighteen patients (22.5%) had a history of other malignancies. Seven patients (8.8%) had previously been diagnosed with breast cancer and 5 patients (6.3%) with colon cancer. A family history of malignancy in a first-degree relative was found in 34 patients (42.5%), with colon cancer being the most common diagnosis (12.5%, *n* = 10). Other malignancies in family histories included breast (10%, *n* = 8), uterus (7.5%, *n* = 6), ovary, prostate, lung, stomach, and brain tumor.

Twelve of the 80 patients (15%) had used hormone-replacement therapy for any duration of time. Eleven patients (13.8%) had a history of oral contraceptive use, and 5 (6.3%) had used tamoxifen. The majority of patients (62.5%, *n* = 50) had a BMI that classified them as overweight or obese. Twenty-five patients (31.3%) reported that they were past or current smokers.

Forty of the 80 patients (50%) had Stage I disease, 13 (16.3%) had Stage II, 12 (15%) had Stage III, and 15 (18.8%) had Stage IV disease (Table 2). The majority of patients (72.5%, *n* = 58) had clear cell histology in combination with serous and/or endometrioid histology. All patients with an endometrioid component had FIGO grade 2 or 3 disease.

 Nineteen patients (23.8%) had positive peritoneal washings (Table 2). Eighteen patients (22.5%) had metastases documented at the time of diagnosis. Twenty-five patients (31.3%) had a focal (<10%) clear cell component within the endometrium. Twenty-two were pure clear cell tumors, and 33 were of mixed histology (Table 2). Thirty-three patients (41.3%) had lymphovascular space invasion. Forty-five patients (56.3%) had lower uterine segment involvement, 25 patients (31.3%) had endocervical involvement, and 10 patients (12.5%) had omental involvement. Thirteen patients (16.3%) had positive lymph nodes.

Radiation was part of the adjuvant treatment for 63 patients (78.8%) (Table 3). All but one of these patients received vaginal brachytherapy (remote after loading ^192^Ir source to a total dose of 21 Gy in 3 fractions or 14 Gy in 2 fractions at 0.5 cm from the vaginal mucosa). One patient was treated only with external beam radiation therapy (EBRT). Five patients received EBRT along with vaginal brachytherapy. One patient received vaginal brachytherapy and whole abdomen radiation treatment with a pelvic boost. Two patients were treated with radiation therapy only at the time of recurrence. Fifty-three patients (66.3%) received chemotherapy (Table 3). Of these, 84.9% (*n* = 45) were treated with a platinum-based regimen, 35 (66%) of whom received carboplatin (AUC = 6) and paclitaxel (175 mg/m^2^) intravenously weekly for 6 cycles. Other regimens used were adriamycin/cyclophosphamide/cisplatin (CAP), topotecan, and weekly paclitaxel. Forty-one of the 53 patients who received chemotherapy also had vaginal brachytherapy.

**Figure 1:**
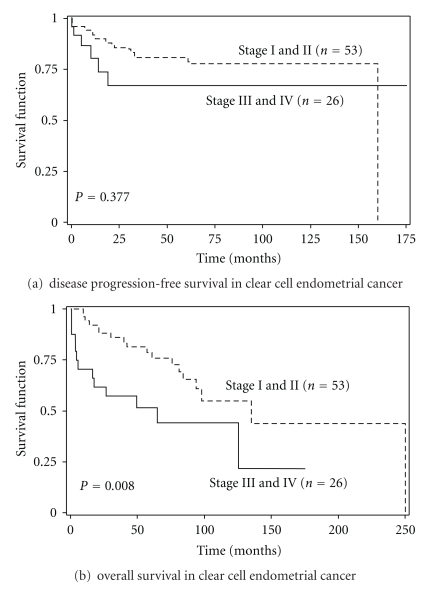
(a) Kaplan-Meier survival curve showing Progression-Free survival in early-stage versus late-stage disease in clear cell endometrial cancer, (b) Kaplan-Meier survival curve showing overall survival in early-stage versus late-stage disease in clear cell endometrial cancer.

Median followup for PFS and OS were 38 months (range: 0 to 175) and 54 months (range: 0 to 250), respectively. For patients with Stage I disease, median followup for PFS was 65.5 months (range: 0 to 160) and for OS was 69.5 months (range: 5 to 250). In Stage II disease, median followup for PFS was 30 months (range: 0 to 142) and for OS was 44 months (range: 10 to 142), and patients with Stage III cancers had median followup for PFS of 15 months (range: 1 to 95) and for OS of 20.5 months (range: 1 to 95). Patients with Stage IV disease had a median followup for PFS of 10 months (range: 0 to 175) and median followup for OS of 27 months (range: 0 to 175 months). At the end of this study, 48 patients (60%) were alive (5 [6.3%] with disease) and 32 patients (40%) had died (Table 4). A total of 17 patients (21.3%) recurred with 4 still alive and 13 succumbing to their disease. PFS was not significantly different between patients with early-stage (Stages I&II) disease and late-stage (Stages III&IV) disease (Figure 1(a); *P* = 0.377). However, OS was significant between these two groups with early-stage disease having a median OS of 135 months (95% CI: 84–250) compared to those with late-stage disease of 65 months (Figure 1(b); *P* = 0.008).

A patient's histology (pure clear cell, clear cell plus serous, or clear cell plus endometrioid) did not have a significant relationship with PFS or OS. In patients with pure clear cell histology (*n* = 22), median PFS was 30 months (range: 0 to 134), and median OS was 43.5 months (range: 1 to 134), while patients with any serous component (*n* = 36) had a median PFS of 32 months (range: 0 to 175) and OS of 47 months (range: 0 to 250). Patients with mixed endometrioid and clear cell histology (*n* = 22) had the best survival, with a median PFS of 61.5 months (range: 0 to 160) and OS of 65.5 months (range: 5 to 237), although this did not reach statistical significance when compared with other histologic subtypes (*P* = 0.21). Lymphovascular space invasion also did not correlate clinically with survival.

Operative notes were available for 72 patients. Of these patients, all but 4 were debulked to no residual disease (*n* = 65) or residual disease of less than 1 cm (*n* = 3). All of the patients with residual disease had Stage IV disease. Their progression-free and overall survivals ranged from 1 to 70 months. When considering all patients, the presence of residual disease had a significant impact on OS (*P* < 0.0001) but not on PFS (*P* = 0.1). The median overall survival in patients with residual disease, even if optimally debulked to less than 1 cm of disease, was 17.5 months versus 135 months in those patients with no residual disease.

There was a significant relationship between age at diagnosis and OS (*P* < 0.001; hazard ratio 1.07; 95% CI 1.03–1.12), independent of FIGO stage. Increased age contributed to shorter overall survival. However, there was no significant relationship between age at diagnosis and PFS (*P* = 0.23; 95% CI 0.98–1.09).

In univariate analysis, vaginal brachytherapy, whether alone or in combination with other radiation therapy, had an impact on OS (median survival with radiation: 140 months versus without radiation: 50 months; *P* = 0.02), but not on PFS (*P* = 0.10). This association was not noted after testing in a multiple regression model. Adjuvant chemotherapy had no significant impact on OS (*P* = 0.26) or PFS (*P* = 0.27). When patients treated with vaginal brachytherapy plus carboplatin and paclitaxel (*n* = 28) were compared to patients who were not treated with this regimen, no significant difference was seen in OS or PFS (*P* = 0.82 and *P* = 0.39, resp.).

## 4. Discussion

Given the rarity of the diagnosis of UCC, meaningful data is lacking in the form of prospective randomized controlled trials. Historically, Abeler and Kjorstad published a chart review of 97 patients with UCC. Although the patients were treated based on different protocols, they found a 5-year survival rate of 42.3% and 10-year survival at 30.9% [10]. No patient with Stage III or IV survived 5 years. Age did not correlate with survival, implying that UCC histology was the most important prognostic factor. Recently, better survival rates of 79% 5-year survival for early disease and 21% for advanced disease have been reported [7].

Fifty percent of our patients presented with Stage I disease, which is higher than usual as most patients present with metastatic disease [5]. This difference may be because the majority of our patients had mixed histology (clear cell plus serous and/or endometrioid) and not pure clear cell carcinoma. Our findings were consistent with a previous publication reporting LVI to have no effect on survival in endometrial cancer [11].

To our knowledge, there are no published studies on the impact of residual disease on survival in UCC. Previous studies have demonstrated a significant survival benefit with optimal cytoreduction at primary surgery in uterine serous carcinoma [12]. When attempting to look at the role of optimal debulking in survival of UCC patients, only 4 patients (5%) in our present study had residual disease of greater than 1 centimeter. While no conclusions can be drawn regarding clear cell histology because of the small number of advanced stage patients in this study, residual disease may be an important factor to consider in future research on survival in UCC patients.

While surgical staging (total hysterectomy, bilateral salpingo-oophorectomy, pelvic and para-aortic lymphadenectomy, omentectomy, peritoneal cavity evaluation with washing, smears, and biopsies of suspicious-looking areas) and optimal cytoreduction are the standard of care in patients with UCC, optimal postoperative management is far from being defined. Treatment strategies vary at different stages. Given the small number of women affected with UCC, factors associated with improved survival are difficult to discern. The Society of Gynecologic Oncology reported that although adjuvant radiation is commonly offered to patients with all stages of UCC, no studies have demonstrated improvements in OS, largely because of lack of power [4]. Studies looking exclusively at UCC are limited [2]. Radiotherapy may be justified given that it may provide improved local control. The present study hypothesizes that there may be an impact on OS when patients received vaginal apex brachytherapy. However, more data is needed to confirm this observation.

No studies have been performed regarding adjuvant chemotherapy in an exclusively UCC population [4, 5]. Several studies examined the role of postoperative chemotherapy in endometrial cancer. The Gynecologic Oncology Group (GOG) 139 trial prospectively evaluated the largest population of pure UCC patients (*n* = 44) for chemotherapy response, although UCC patients only comprised 3.7% of the total study population [13]. Clear cell histology was a negative predictor for PFS and OS compared to other histologic subtypes. Carboplatin and paclitaxel have some efficacy in women with UCC with acceptable toxicity [14]. Although other chemotherapeutic regimens have been examined, their efficacy in the UCC subset of patients has yet to be established [15]. A prospective cohort study of 22 patients with Stage I UCC and uterine serous carcinoma concluded that adjuvant therapy may not be necessary in early-stage patients after surgical staging [16]. Given the data available, the authors believe it is reasonable to offer all patients with UCC adjuvant chemotherapy, although the best therapeutic regimen and its true benefit in Stage I patients are still up for discussion.

The potential association of endometrial cancer with other malignancies, particularly carcinomas of the breast and colon, has been investigated previously [17]. A study comparing the rate of breast cancer in patients with uterine serous cancer and that in patients with endometrioid endometrial cancer demonstrated that 19.4% of serous patients had a history of breast cancer compared to 3% of those with endometrioid histology [18]. In our study, 8.8% of our patients had a personal history of breast cancer. This disparity in breast cancer rates between uterine serous cancers and UCC may be due to differences in breast cancer treatment and rates of tamoxifen use or more likely, due to the fact that many of our patients had mixed histology. Women with Lynch syndrome have a 20–60% lifetime risk of developing endometrial cancer [19]. In fact, many women with Lynch syndrome present with endometrial cancer as their first malignancy [20]. There is a higher incidence of nonendometrioid endometrial cancer in patients with Lynch Syndrome (43%) than in those with sporadic uterine tumors, despite younger mean age at diagnosis. In one recent study of patients with endometrial cancer and Lynch syndrome, the authors reported that 21% of the patients had clear cell cancer [19]. Five patients (6.3%) in our study had a personal history of colon cancer, one of whom had a sister with colon cancer. An additional five patients had a first-degree relative with colon cancer, without a personal history of gastrointestinal malignancy. Providers should keep in mind that patients diagnosed with UCC (particularly at a younger age) may carry the Lynch syndrome mutation.

## 5. Conclusions

This retrospective, single-institution report of uterine clear cell cancer demonstrates that most patients with UCC have clear cell histology in combination with serous and/or endometrioid histology. In our present study, we show that age and early-stage versus late-stage disease have a significant impact on overall survival. Of particular interest, vaginal brachytherapy also had an impact on overall survival regardless of other aspects of a patient's treatment. When comparing this data with data from patients with uterine serous cancer from the same institution, there does not seem to be a great difference in recurrence rates between clear cell and serous histologies. In a study by Kelly et al., 28% of Stage I serous cancers recurred, and in our study 20% of Stage I UCC patients recurred [21]. As with all research on this aggressive histologic subtype of endometrial cancer, our data is limited by small sample size. However, these results may help guide future prospective research from which we draw more concrete conclusions regarding treatment and followup of patients with UCC.

## Figures and Tables

**Table 1:** Patient demographics.

Characteristic	Number of patients	Percent
Age at diagnosis (years)		
40–49	5	6.3
50–59	13	16.3
60–69	30	37.5
70–79	16	20.0
80–89	15	18.8
≥90	1	1.3
Race		
Caucasian	69	86.3
African-American	4	5.0
Unknown	7	8.8
Positive medical history		
Obesity	50	62.5
Hypertension	46	57.5
Diabetes	22	27.5
Coronary artery disease	14	17.5
Breast cancer	7	8.8
Colon cancer	5	6.3
Other malignancy	6	7.5
Past or current tobacco use	25	31.3
Medication use (past or current)		
Hormone replacement therapy	12	15.0
Oral contraceptives	11	13.8
Tamoxifen	5	6.3

**Table 2:** Histopathologic findings.

	Number of patients	Percent
Stage (FIGO^a^ 1988)		
I	40	50.0
II	13	16.3
III	12	15.0
IV	15	18.8
Histology		
Pure clear cell	22	27.5
Clear cell + serous ± endometrioid	36	45.0
Clear cell + endometrioid (no serous component)	22	27.5
Washings		
Positive	17	21.3
Negative	61	76.3
Not done	2	2.5
Lymph nodes		
Positive	13	16.3
Negative	67	83.8

^a^International Federation of Gynecology and Obstetrics.

**Table 3:** Adjuvant treatment.

	Number of patients	Percent
Radiation	63	78.8
VB^a^ only	56	70.0
VB + EBRT^b^	5	6.25
EBRT only	1	1.25
VB + WART^c^	1	1.25
Chemotherapy	53	66.3
Carboplatin & Paclitaxel	35	43.75
Cyclophosphamide, Adriamycin, & Cisplatin	10	12.5
Other (inc. topotecan, weekly paclitaxel)	8	10.0

^a^VB: vaginal brachytherapy; ^b^EBRT: external beam radiation therapy; ^c^WART: whole abdomen radiation therapy.

**Table 4:** Patient outcomes.

	Number of patients	Percent
Alive no evidence of disease	43	53.8
Alive with disease	5	6.3
Dead of disease	21	26.3
Dead no evidence of disease	10	12.3
Lost to follow-up (disease status unknown)	1	1.3
